# Use of artificial intelligence in analytical systems for the clinical laboratory

**DOI:** 10.1155/S1463924695000010

**Published:** 1995

**Authors:** John F. Place, Alain Truchaud, Kyoichi Ozawa, Harry Pardue, Paul Schnipelsky

**Affiliations:** 1 DAKO A/S Produktionsvej 42 Glostrup/Copenhagen 2600 Denmark

## Abstract

The incorporation of information-processing technology into analytical systems in the form of standard computing software has recently been advanced by the introduction of artificial intelligence (AI), both as expert systems and as neural networks.

This paper considers the role of software in system operation, control and automation, and attempts to define intelligence. AI is characterized by its ability to deal with incomplete and imprecise information and to accumulate knowledge. Expert systems, building on standard computing techniques, depend heavily on the domain experts and knowledge engineers that have programmed them to represent the real world. Neural networks are intended to emulate the pattern-recognition and parallel processing capabilities of the human brain and are taught rather than programmed. The future may lie in a combination of the recognition ability of the neural network and the rationalization capability of the expert system.

In the second part of the paper, examples are given of applications of AI in stand-alone systems for knowledge engineering and medical diagnosis and in embedded systems for failure detection, image analysis, user interfacing, natural language processing, robotics and machine learning, as related to clinical laboratories.

It is concluded that AI constitutes a collective form of intellectual propery, and that there is a need for better documentation, evaluation and regulation of the systems already being used in clinical laboratories.


Journal of Automatic Chemistry, Vol. 17, No. 1 (January–February 1995), pp. 1–15

### Use of artificial intelligence in analytical systems for the clinical laboratory

**John F. Place, Alain Truchaud, Kyoichi Ozawa, Harry Pardue and Paul Schnipelsky**
*IFCC Committee on Analytical Systems: Chairman A. Truchaud*

*The incorporation of information-processing technology into analytical systems in the form of standard computing software has recently been advanced by the introduction of artificial intelligence (AI), both as expert systems and as neural networks.*

*This paper considers the role of software in system operation, control and automation, and attempts to define intelligence. AI is characterized by its ability to deal with incomplete and imprecise information and to accumulate knowledge. Expert systems, building on standard computing techniques, depend heavily on the domain experts and knowledge engineers that have programmed them to represent the real world. Neural networks are intended to emulate the pattern-recognition and parallel processing capabilities of the human brain and are taught rather than programmed. The future may lie in a combination of the recognition ability of the neural network and the rationalization capability of the expert system.*

*In the second part of the paper, examples are given of applications of AI in stand-alone systems for knowledge engineering and medical diagnosis and in embedded systems for failure detection, image analysis, user interfacing, natural language processing, robotics and machine learning, as related to clinical laboratories.*

*It is concluded that AI constitutes a collective form of intellectual property, and that there is a need for better documentation, evaluation and regulation of the systems already being used in clinical laboratories.*

### THEORY

### Introduction

Automation was introduced fairly late in the field of clinical chemistry. In the 1930s, increasing use was made of simple instruments based on classical principles of analytical chemistry. Instrumental analysis became more important because of a switch to physical and physico-chemical measurement approaches. The latter methods were often more sensitive and could be used to quantify smaller amounts of analytes; they were frequently simpler and faster than more conventional methods [1]. Spectrometers, flame spectrometers, instrumentation using electrochemical electrodes and instrumental separation methods (centrifugation, electrophoresis) were particularly important in this respect.

Until 1950 it was still common for clinical chemists to do determinations manually using instruments for the measurement step only. The mechanization of sample-processing functions was greatly influenced by the segmented flow system started with the auto-analysers of Skeggs [2], that were made commercially available at the end of the 1950s. This process has continued through many phases with the most recent advances being in the area of robotics [3].

Mechanization and automation of most of the functional components of analytical systems [4] has allowed clinical chemistry to grow and develop into a systems approach in which all aspects of clinical determinations have become integrated. Software, that was originally used primarily to sequence the mechanical processes and process raw measurement data, has become so sophisticated and generally available that it can now be used to monitor and control processes and to interpret information. This development has made it possible to incorporate intelligence into advanced analytical instrumentation. This form of machine intelligence is also called Artificial Intelligence (AI). An attempt is made later in this paper to define intelligence, and examples of intelligent systems will be given.

AI is being used increasingly in the clinical laboratory, both in the form of stand-alone expert systems for clinical decision making and as knowledge-based systems embedded within laboratory instrumentation. In addition to AI systems that can be implemented in standard computers, progress is being made in the development of software architectures that mimic human intelligence. Research in this area is leading to a better understanding of human (natural) intelligence. This approach is so different that it is sometimes termed 'naturally intelligent' to distinguish it from other artificial intelligence approaches [5]. In this paper no such distinction will be made and all machine intelligence will be described as artificial.

Despite these advances, or perhaps because of them, the general level of understanding of AI is very low; users are not aware of what AI really is and what it can or cannot do. One of the main reasons for this is that the terminology is confusing and even ambiguous and that the concept of AI for many users has overtones of science fiction. The aim of this paper is to attempt to present the concepts and potential applications of AI in a format that is more easily understood by clinical chemists.

#### *Automation*

The earliest forms of automatic machines had mechanical forms of feedback and were capable of repeated action: an example is a mechanical clock. Technically, this type of system is called an automaton. With the development of convenient sources of power, automation was expanded during the Industrial Revolution.

*Correspondence to John F. Place, DAKO A/S, Produktionsvej 42, 2600 Glostrup/Copenhagen, Denmark.*

© 1995 The exclusive copyright in any language and country is vested in the International Federation of Clinical Chemistry (IFCC).

The most significant advances that have facilitated the evolution of automation in more recent years include the development of electronics and digital computing, advances in software storage capacity and in sensor technology and the derivation of theories of control systems including AI.

### System operation and control

Before trying to define intelligence and the position of AI in the clinical laboratory, and in order to place AI in a wider perspective, it is useful to look at the higher level functional processes of system operation and control in clinical laboratory automation. These are illustrated in figures 1 to 4.

Figure 1 shows the functional elements of an instrumental component such as the incubation block of a clinical instrument. In this example the electrical elements in the incubation block (the effectuators) produce heat and warm the block (output). Heat dissipates through the block (feedback) and the temperature of the block (input) is measured by a built-in thermocouple and compared with a set of standards. When the temperature rises above a set value, power to the heating element is switched off. The block cools by loosing heat to its surroundings. When the temperature falls below a set value, power is applied to the heater again until the measured input value again rises above the set value. This sort of feedback system is a central idea in cybernetics, defined by the American mathematician Norbert Wiener in 1947 as 'the science of communication in the animal and in the machine'.

In figure 2 the elements from input to output are summarized in the single function 'process' and feedback is generalized into the wider term 'control'. Any form of automation or decision-making system can be regarded very simply as an input giving rise directly or indirectly via a process system to an output, which then gives rise to some feedback or control.

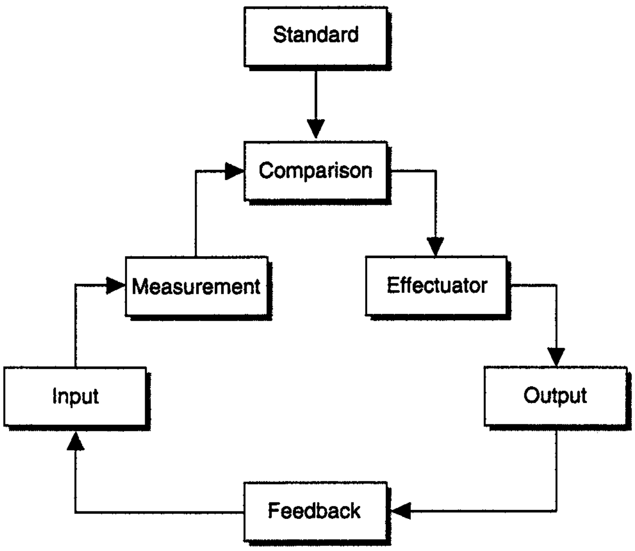

*Figure 1. Functional elements of an instrumental component.*

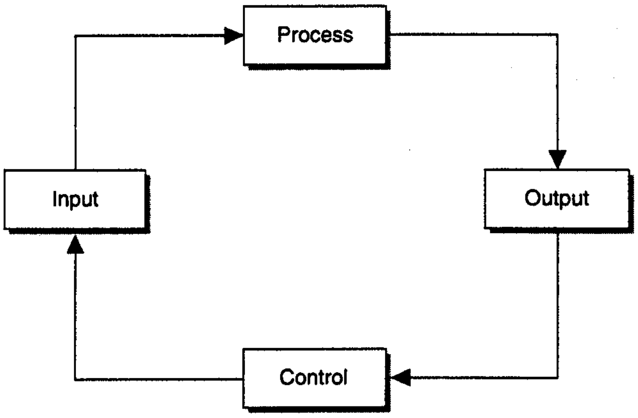

*Figure 2. The control loop in process control.*

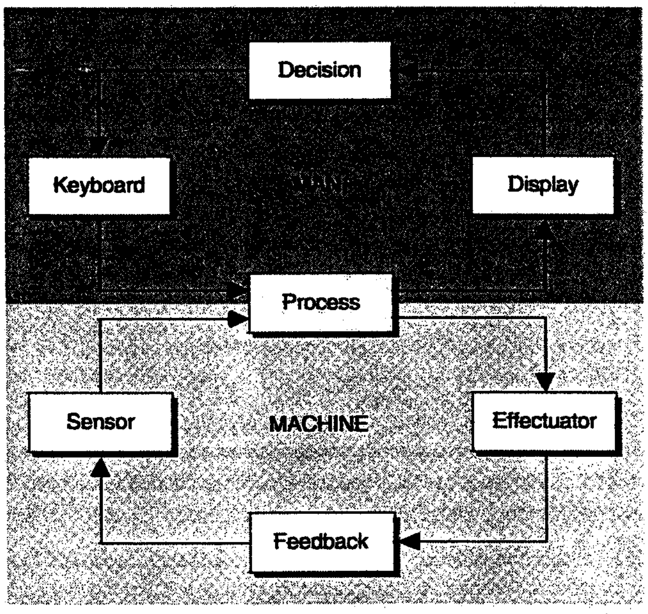

*Figure 3. The man-machine interface and process control.*

Figure 3 introduces the concept of decision as a mainly human control function and feedback as a mainly machine control function. As in figure 2, the input is on the left-hand side of the figure and the output on the right-hand side. This figure also illustrates the difference between a system that is embedded within an instrument

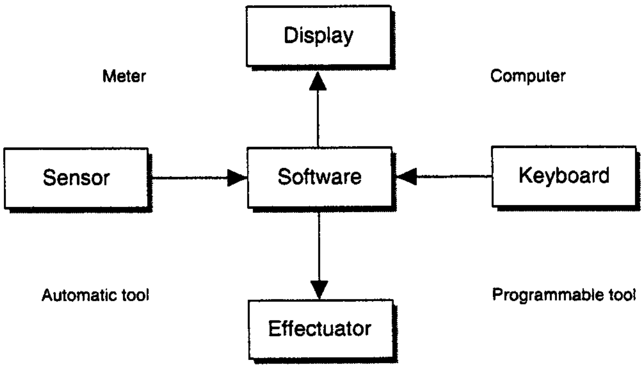

*Figure 4. Classification of simple instruments into four categories.*

(bottom half of the figure) and the open or stand-alone system (total figure). In the stand-alone system, direct and interactive human control is possible as output is displayed for human evaluation. In the embedded system, cycles of processes are evaluated automatically by intelligent controllers below the surface of the man-machine interface.

On the basis of whether the input is from man (for example via a keyboard) or machine (by sensor and associated circuitry) and whether output is to man (for example as a display) or to machine (by effectuator), one can develop a basic classification of simple instruments. This is illustrated in figure 4. In a meter, the machine (sensor) input provides data that are processed by software and relayed as information to a display, while in a personal computer it is the human operator (for example via a keyboard) that provides input to the software. Effectuators (for example a motor or some other form of tool) can be monitored automatically by a sensor system (for example in robotics), or programmed via a keyboard by a human operator.

Figure 4 also shows that software is the central feature of processing and control in automation. This is of course a grossly simplified illustration. Real instrumentation is very complex, integrating many such simple units into a complex whole.

### Intelligence and learning mechanisms

With the introduction of programmable software it became possible to introduce great flexibility into the system, both in the operational processes and in the feedback loop. As more flexibility and higher level control is introduced into the system it can acquire the capacity to become 'intelligent'.

However, it is not always possible or appropriate to introduce machine intelligence into system operation and control. When the decision sequence is clear, an algorithm can be defined, and a conventional software programme may be more appropriate.

Intelligence is very difficult to define. It has something to do with the faculty of understanding and the capacity to know or to learn and much of the basic work on AI is to do with the mechanisms of learning. The major advantage of AI is that it allows learning via the accumulation of knowledge.

However, learning includes a large proportion of insight characterized by good classification of the stimulus and 'silent' trial and error to reinforce the response.

### Knowledge engineering

In an attempt to get away from science fiction connotations, AI technologies are now commonly termed 'knowledge engineering' and the intelligent computer software that embodies knowledge is called the 'expert system'. Because it has been rather difficult to develop practical applications of automatic learning, expert systems often do not include the ability to learn by themselves. Nevertheless, such expert systems are able to make decisions based on the accumulated knowledge with which they are programmed and are therefore commonly included within the definition of AI systems. An excellent introduction to expert systems is given by Bartels and Weber [6], and Wulkan [7] provides a recent review of the state of the art in relation to clinical chemistry.

Expert systems possess high-quality, specific knowledge about some problem area and build upon the problem-solving capabilities of human experts. Human expertise is characterized by the fact that it is perishable, difficult to transfer and to document, hard to predict and expensive to obtain, and one of the main aims of expert systems is to overcome these frailties by acquiring, representing, documenting and preserving expertise in a form that then becomes widely available at lower cost. The very process of realizing and documenting this human expertise may well improve upon it, and in some cases, expert systems use exhaustive or complex reasoning that individual human experts would be incapable of using. However, the expert system, like its makers, is not necessarily infallible.

Expert systems, like their human counterparts, should provide a high level of skill with adequate robustness. Unlike most conventional programs, expert systems usually have the ability to work with represented and uncertain knowledge and the ability to handle very difficult problems using complex rules. On the other hand, if the problem requires creative invention, it is really not possible to develop an expert system to solve it. Similarly, if the solution requires a significant input of general broad common-sense knowledge, the expert system may not be able to deal with the problem in a satisfactory way. Differences between conventional software programs and expert systems are listed in table 1. Some of the terms used in table 1 are discussed below under the section 'the knowledge base'.

The distinction between a conventional software program and an expert system may not be as clear as table 1 might suggest. Not all existing expert systems are able to deal with uncertainty and some operational expert systems use conventional Bayesian probabilistic methods to deal with

*Table 1. Conventional programs and expert systems compared.*

| Conventional programs | Expert systems |
|---|---|
| Represent and use data | Represent and use knowledge |
| Algorithmic | Heuristic |
| Iterate repetitively and exhaustively | Use inferential processes |
| Use a decision sequence | May have numerous ways to reach conclusion |
| Must have all decision rules | |
| Stop if data missing | Allow for missing information |
| Derive one solution consistently | Derive best decisions possible |
| Use numbers and equations | Can also use concepts |
| Present a result with confidence limits | Provide uncertain results |
| | Rank results by likelihood |
| Appropriate for known, accepted ways to solve a problem, especially with only one solution | Appropriate for imprecise information and conceptual problems |

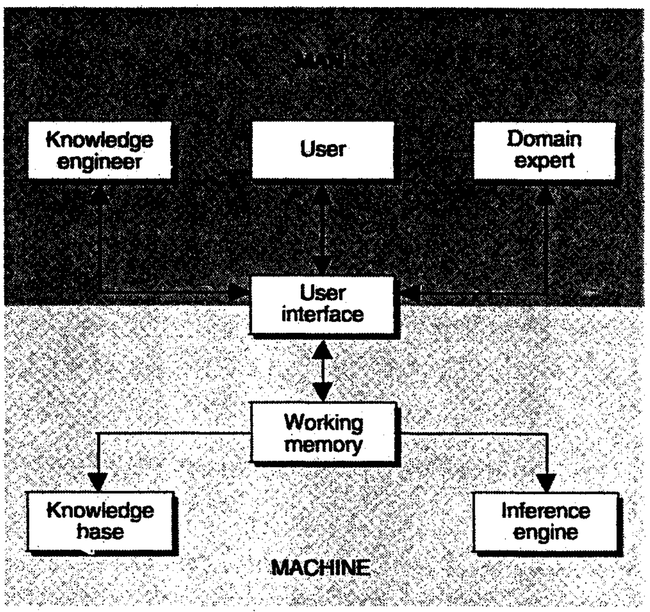

*Figure 5. Elements of an expert system.*

uncertainty. A complex AI system may also contain a series of conventional programs embedded within it.

An expert system consists of at least four structural components (figure 5): the *knowledge base* that contains the expert knowledge (the facts), the rules and the heuristics, i.e. experience and as much of the intuition as can be represented; the *inference engine* that contains the reasoning and problem solving strategies; the *working memory* that holds data about the particular situation the system is evaluating and records the steps of the reasoning sequence; and the *user interface* by which the user (or another expert system, or the main memory of an information system) can communicate with the expert system. An expert system without the knowledge base is called a 'shell'.

The expert system may have additional modules, for example one that can provide the user with an explanation of how and why it arrived at the conclusion, or a learning module with evaluation and feedback systems that can update data and even the rules of the expert system.

There are two components in expert systems that the reader may not be familiar with—the knowledge base and the inference engine. These are described in the following sections.

### The knowledge base

The knowledge base is developed by the knowledge engineers who query the domain experts. An important distinction is made between data (which consist of numerical representations, equations and algorithmic procedures) and information (which encompasses facts, concepts, ideas, previous experience and rules of thumb). The knowledge engineer feeds the system base with details from manuals and test procedures etc. (facts) and interviews the domain experts to learn how and when they reach a decision (rules) and to define the way experience and intuition play a role (heuristics). The way in which knowledge engineers interpret real-world knowledge in terms of computer data structures is a critical component of an AI system and is called 'knowledge representation'.

*Table 2. Representations used in MYCIN.*

| | |
|---|---|
| 'Definitely not' | = −1·0 |
| 'Almost certainly not' | = −0·8 |
| 'Probably not' | = −0·6 |
| 'No evidence' | = −0·2 to 0·2 |
| 'Slight evidence' | = 0·3 |
| 'Probably' | = 0·6 |
| 'Almost certain' | = 0·8 |
| 'Definite' | = 1·0 |

A major characteristic of expert systems is their ability to accept uncertain data and to work with concepts. There are various sources of this uncertainty of information. A variety of techniques can be applied to allow uncertain facts and imprecise knowledge to be included and to give the software the ability to work with both quantitative and qualitative information.

Uncertainty of facts arises for example in sampling a population. The uncertainty of sampling is a known statistical quantity and can be dealt with using classical statistical methods.

Uncertainty also arises in the reasoning processes in which the same data can give rise to several alternative conclusions or in vagueness of description, so that different observers will classify their observations in different ways, giving rise to 'fuzzy sets'. Fuzzy sets are a particular branch of mathematics founded in 1965 by Lofti A. Zadeh, now Professor of Computer Sciences at the University of California at Berkeley. Fuzzy logic is so important that the Japanese Industry Ministry, together with the Education Ministry and private industry have set up a Fuzzy Logic Institute in Fukuoka Province.

Early expert systems used certainty factors to deal with this sort of uncertain information. MYCIN [8], one of the earliest expert systems in the clinical disciplines and originally developed to recommend antibiotic therapy for bacterial infections of the blood, used the representations shown in table 2, with certainty factors scaled from −1.0 to +1.0.

Expert systems can deal with concepts by defining them using attributes and then representing them in the form of symbols, frames, rules etc. For example in symbolic processing, the symbol can be a string of characters (for example words describing the concept) or a bit pattern (for example an icon on a computer screen). These symbols can then be manipulated by matching them, assembling sets etc. and subjected to straightforward logical decisions and heuristic operations. For the reasoning capacity itself, it is immaterial what the knowledge representation instance (symbol, frame, rule etc.) stands for and hence what the concept actually is.

### Knowledge representation

Several basic knowledge representation techniques are known: these are listed in table 3. Fact lists are the simplest

*Table 3. Knowledge representation techniques.*

| | |
|---|---|
| * | Facts list<br>—no uncertainty |
| * | Symbols<br>—representing defined concepts<br>—uncertainty included in the definition |
| * | Production rules<br>—premise + action part<br>—certainty factor given to conclusion |
| * | Structured objects and object-oriented structures<br>—descriptive statements<br>—symbolic, used in morphology and anatomy |
| * | Associative networks<br>—entities (nodes)<br>—relationship between entities (arcs) |
| * | Frames<br>—complex nodes allowing data entry<br>—contains slots with attributes<br>—organized in class hierarchy |

form of knowledge representation. They are used to list data and equations and the uncertainty attached to the facts can be precisely defined. Symbols are used to define items or concepts. As described above, their uncertainty lies in the definition itself and not in the symbol.

Production rules consist of a set of preconditions (the premise) and an action part that leads to a conclusion (for example, see O'Connor and McKinney [9]). If the premise is true, the action part is justified and a certainty factor is given to the conclusion.

Structured objects are used, for example by Bartels *et al.* [10], in morphological or anatomical representation that are best dealt with in the form of descriptive, declarative statements. In associative (or semantic) networks both the entities themselves and the relations between the entities are represented, the former by nodes and the latter by arcs connecting these nodes.

Frames are analogous to complex nodes in a network (for example Du Bois *et al.* [11]). They allow a more precise and comprehensive description of an entity. In a frame, a data object has slots that contain attributes of the object and a slot can activate data gathering procedures if the information it contains is incomplete. Frames are usually organized in a class hierarchy with information in higher classes inherited by lower classes. Each problem requires its own knowledge representation and there seems to be no consensus concerning the use of the various knowledge representation techniques. Hybrid techniques abound. An example of a hybrid frame and rule architecture is given by Sielaff *et al.* [12] in ESPRE, a knowledge-based system to support decisions on platelet transfusion.

### Inference engine

The inference engine implements the problem-solving strategies built up by the knowledge engineer. A common technique in developing the expert system is to identify the 'primitives' or independent variables first and then devise a system in which these can be redefined to represent higher level or dependent knowledge. Each knowledge based system is thus built of 'primitive' knowledge bases linked by nodes to higher level knowledge bases. At each node, a reasoning process is designed and all the reasoning procedures together form the inference engine. The inference engine is independent of the contents of the knowledge base.

The way in which the inference engine reasons can be illustrated by looking at a typical rule. The condition part (the premise) has three components, the object, a logic operator and an attribute. The action part then specifies what will be done if the condition part is found to be true; for example the rule;

IF (nuclear size is $>40\ \mu m^2$)
THEN (compute the total absorbance [A]).

At the start, the system is pointing at the location where the input 'nuclear size' is written into the working memory. As this follows an IF statement it is identified as a component of the condition part of the rule. The object 'nuclear size' is matched with an object table. The logical operator 'is > ' is identified and the value to be attributed is requested from the user. The value is entered through the user interface into the working memory. The inference engine retrieves this value and compares it with the value of $40\ \mu m^2$ specified in the rule. If the rule is triggered, the action part is interpreted by the inference engine and output is provided to execute the THEN part. Finally, the inference engine writes the completed sequence into the working memory, to add to the session history.

It is well known that the higher level of performance of experts in any domain is related to their ability to make a structured analysis, rather than trying all the alternatives. Especially in medical diagnosis, powerful hierarchical structures lead from symptoms to diagnosis.

The inference engine typically operates using two sets of rules, the first concerned with the search space(s) among knowledge representation instances within which to look for solutions and the second concerned with which nodes to evaluate and in which order.

There are two basic ways of searching. If the routing decisions at each node are highly certain and unambiguous, a depth first strategy is indicated. If such a strategy turns out to give the wrong answer, the program returns to the start and follows a new in-depth search. In the breadth first strategy the search space is searched exhaustively at each level to avoid going deep into a blind path. A reasonable (in relation to time taken and use of memory space) focus on the problem is retained in a so-called bounded, depth-first strategy.

Once the strategy is indicated, the inference engine will compare observed facts and rules, keep a record of the sequence and outcome of rule use, resolve conflicts between rules, present the user with a set of alternative results including degree of certainty and with a detailed explanation of the reasoning sequence. It is important to stress that problem-solving strategies are heavily dependent on the representation and inappropriate representation can make an expert system fail.

A number of inference (problem-solving) mechanisms can be recognized and some are listed in table 4.

*Table 4. Some inference (problem-solving) mechanisms.*

| | |
|---|---|
| * | Heuristic search<br>—rules of thumb |
| * | Generate/test<br>—defining search spaces as required |
| * | Forward/backward chaining<br>—pruning decision trees |
| * | Recognize/act<br>—special conditions requiring specific actions |
| * | Constraint directed search<br>—main skeleton of acceptability is known |
| * | Metarules<br>—exhaustive mapping |

Heuristic searching is based on experience or 'rules of thumb'. Some people, for example in maintenance and failure detection, are able to diagnose problems more quickly and effectively than others, because of domain-specific knowledge acquired as a result of experience. The search space for a particular problem can be rapidly narrowed down by this sort of knowledge.

In some cases the search space is not provided, but generated as the system proceeds and evaluated shortly thereafter. This is known as 'generate/test'.

Forward chaining is testing preconditions for the truth of a given hypothesis for given data, rather like pruning a decision tree. Forward chaining is useful when the number of possible outcomes is limited.

Backward chaining, with the system seeking data to justify the selected hypothesis, is the reverse and is more appropriate when there are a large number of possible outcomes. Should a hypothesis turn out to be false, the system can undo conclusions that led to or followed from the false hypothesis.

Some sets of features that occur during problem solving require specific actions. These are solved usually by IF (features pattern) THEN (take action) rules. This type of inference mechanism is called 'recognize/act'.

In a constraint-directed search the main skeleton of what is acceptable is known, but not exactly what it is.

Metarules are part of the procedural knowledge of expert systems. They are used to evoke programs and to guide the inference process and thus define how to use the rules in the system. They are basic blocks of (usually) IF_THEN rules for exhaustive mapping.

The inference engine may make use of one or a number of these problem-solving mechanisms.

### Problems with expert systems

Since the expert system is built using knowledge from the expert, it may perform more or less like an expert. However, as an expert system does not get tired, it will give more consistent results than its human counterparts. A good expert system may even give better results than human experts performing at their best.

Unless knowledge already exists and is accepted (for example in the form of maintenance manuals), knowledge acquisition, i.e. what to put into the knowledge base and more particularly what to leave out, is a problem. It may be difficult to ensure that all the relevant knowledge has been obtained from the human expert and indeed the knowledge engineer may be in doubt about what is relevant.

Domain experts will always provide their knowledge in a specific context, whereas the expert system attempts to apply this knowledge to all cases. Knowledge acquisition tends to increase the size of the knowledge base dramatically. For example in a thyroid report interpretation expert system, the knowledge base doubled in size to move from 96% to 99.7% agreement with experts (Compton [13]). For this reason the function of a new expert system should be carefully defined. If the expert system is to be used for teaching, it will contain a large amount of explanatory functions that are not needed if the expert system is to be used to provide expert judgements in consultation. If the system is to be used to screen input and select cases that need detailed attention, its knowledge base should be broad, but without great depth. On the other hand, if the system is to function as an expert in complicated cases, the knowledge base should be large but limited to a defined field of expertise.

One of the most important features of an expert system is that it can be improved by the addition of further information from users. Provision should be made for maintaining the knowledge base. Because of rule overlaps and the complex interrelationships between rules, maintaining the knowledge base can become complex. Tools exist for knowledge base validation, test and maintenance. Guidelines and references are given by Pau [14].

A further question is one of acceptance and compliance. Will the intended users enter the (correct) information requested by the expert system and will they accept the advice offered by the expert system? Many medical diagnostic expert systems seem to fail to acquire common use because they do not address these sorts of problems.

### Programming languages and hardware

The first hardware architecture widely used in computers consisted of a central processor connected by a single data bus to memory. The processor, acting on instructions from the software program stored in its memory, dictates what is to be done at any given time, sends data to and receives data from memory and performs the bit operations that are the basis of computations and logic operations at the machine (semi-conductor) level. As well as holding the stored program, the memory stores initial data and intermediate and final results of computations. Even very complex computers still build on this simple (von Neumann) architectural unit.

As the system becomes more complex, external memories are added, together with input/output units to allow the system to communicate with the outside world. This requires an increase in the number of pathways for

*Table 5. Computer languages.*

| Generation | Characteristics | Examples |
|---|---|---|
| 6 | Object-oriented | Smalltalk, C++ |
| 5 | Variable unification backtracking | Prolog, LISP |
| 4 | Recursion | Pascal, Modula, ADA, C |
| 3 | Parameter procedures | Fortran, Cobol |
| 2 | Macros, 'go to' | Basic |
| 1 | Machine code | Assembler |

communication. Digital signals representing memory locations, control instructions and data are passed along such buses. An internal clock is required to pace the operations and to ensure that all the parts of the computer stay synchronized. In fact software control and synchronization (housekeeping functions) become a major concern as complexity increases, mainly because the computer operates in a series of sequential steps and each operation must wait for its predecessors to be completed.

Many computer languages (table 5) have been developed over the years to deal with this increasing complexity. They range from the machine-code languages, like Assembler, to object-oriented languages, such as Smalltalk. Most of these computer languages have been largely constrained by the von Neumann computer architecture and have been written for serial processing.

The increase in computer capacity building on the von Neumann architecture has been made possible by the development of surface (quasi two-dimensional) technology in the 1970s and 1980s. As a result, computing capacity per dollar has been able to double every three years since the early 1950s [15]. At first it looked as though the increase in hardware capacity would meet the increased demands for power and speed that would be needed to solve real problems. However, in 1973, Sir James Lighthill suggested that real problems would require too great an increase in the power and speed of conventional computer hardware to be feasible [16]. He called this the 'combinatorial explosion'. Building intelligence on the base of slavish searching through data by serial processors was just not practical—especially not for problems like pattern recognition that characterize intelligence.

The human brain does not succumb to this combinatorial explosion in order to identify, for example, a face. In fact, we find it relatively easy to do this, while artificial image recognition systems find this task particularly difficult.

Some capacity problems can be solved by parallel processing. Human intelligence is of a concurrent nature and therefore many applications of artificial intelligence concern processes that occur concurrently and are best solved by processing in parallel. Actually, it is possible and not unusual to emulate parallel processing using serial processors, but this requires extremely complex control mechanisms to interrupt and synchronize the concurrently running processes.

Attempts have been and are being made to design systems more suited to solving the mixed sequential and concurrent nature of real world problems, in which the software and hardware architectures are superimposable. One such design is the transputer chip designed to run the software language Occam [17]. The transputer is a single chip computer with a processor, local memory, reset, clock and the necessary interfaces for communication. Such a chip avoids most of the communication problems of conventional computers and can be built into arrays and networks that resemble more closely the sort of structure found in the human brain. The Occam language has an architecture that corresponds to the transputer so that hardware and software is compatible in a structural way. Theoretically, at least, this makes the system more robust and would allow it to continue to perform even if a part is removed.

### Neural networks

The human brain does not solve problems by using a well-defined list of computations or operations. Decisions are based almost exclusively on what has been learned by experience. The concept of modelling a system on the structure of the human brain was introduced by McCulloch and Pitts as the neural model in 1943 [18]. In neural networks (figure 6), individual processing elements communicate via a rich set of interconnections with variable weights. Just like biological neurones, processing elements exist in a variety of types that operate in different ways. One or more inputs are regulated by the connection weights to change the stimulation level within the processing element. The output of the processing element

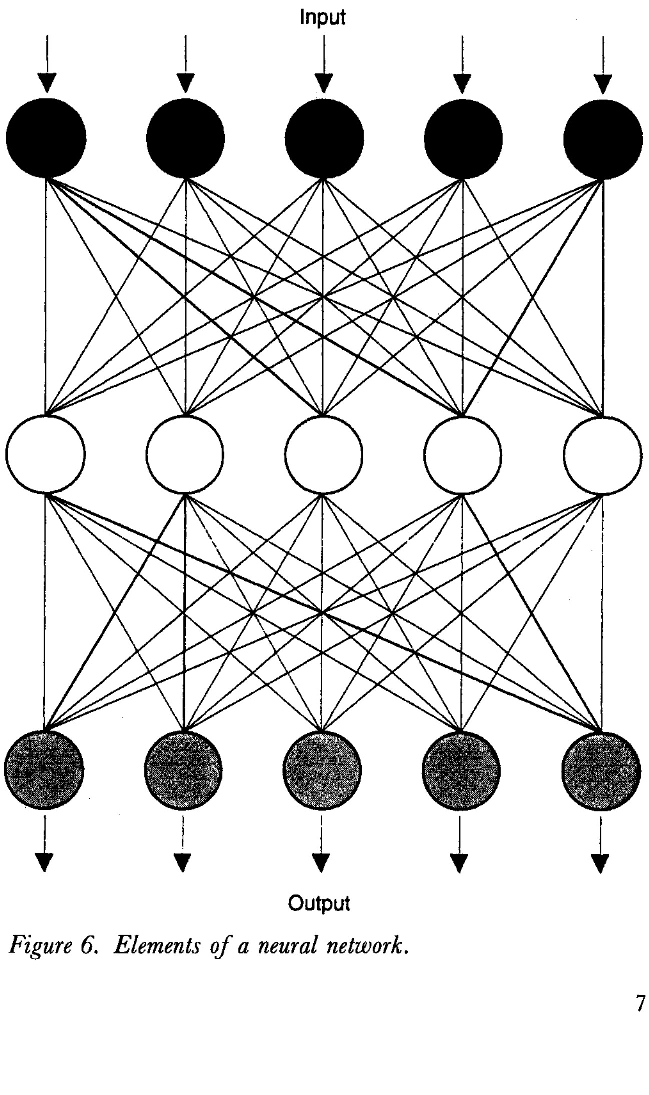

*Figure 6. Elements of a neural network.*

is related to its activation level and this output may be non-linear or discontinuous. The network is taught rather than programmed and teaching results in an adjustment of interconnect weights, depending on the transfer function of the elements, the details of the interconnect structure and the rules of earning that the system follows. Thus memories are stored in the interconnection network as a pattern of weights. Information is processed as a spreading changing pattern of activity distributed across many elements. A short review of neural networks and their potential applications in biotechnology is given by Collins [19]. This includes a table of software tools that are available for neural net development.

The neural network is thus a third type of information-processing technology (the other two being conventional software languages and expert systems). The neural network is good at doing the sort of things the human brain is good at, in particular pattern classification and functional synthesis. These will be described in the section on applications. The neural network acts as an associative memory, it is good at generalizing from specific examples, it can tolerate faults in the network and it can be self-organizing. Differences between neural networks and expert systems are given in table 6.

In theory, part of the intelligent performance of neural networks relies on the physical structure of the network. In practice, neural networks are usually simulated in computers using conventional hardware, so that the software structure and the hardware structure are not physically related.

Constructing networks in hardware is a problem. The reason is that semiconductor technology is essentially two dimensional, whereas the physical embodiment of neural networks is basically three dimensional. All integrated circuits become problematic when they are miniaturized. Connections act as antennas over minute distances leading to cross talk; electrical signals are slowed by inductance and capacitance; the dwell time of electrons passing rapidly may not be sufficient to effect a change in the circuit; there may be problems with the random distribution of circuits on the microscopic scale; electrons may even cause breaks by electromigration of atoms. Apart from this there are significant problems with the slow speed of off-chip connections and physical limits to the number of connections that can be made to a chip.

*Table 6. Expert systems and neural networks compared.*

| Expert system | | Neural network |
|---|---|---|
| Software defined | | Hardware defined |
| Knowledge base | Structure | Processing elements |
| Inference engine | | Interconnections |
| Standard computer (transputer) | Memory | Interconnect weight pattern |
| Programmed | Learning | Trained<br>Can generalize |
| Depends on knowledge engineer | Action mode | Processes time-dependent spatial patterns<br>Self organizing |
| Small tolerance | Faults | Very high tolerance |

Such physical constraints of structure have led researchers to look for alternatives to the semiconductor approach to hardware. Optical signals have several advantages over electrical signals as a basis for computing. For example, they may allow speeds three orders of magnitude faster. Light beams do not need carriers and can even cross without interacting and are thus more amenable to three-dimensional structures. The main limitations to this approach are related to the materials used. Some progress has been made, for example using holographic images as the connectors between opto-electronic neurones in photoreactive crystals [20], still optical computing is probably more science fiction than fact. Attempts are also being made to develop hardware on a biological or molecular basis.

### A model of human intelligence?

While the expert system can justify its reasoning strategy, declaring which search space was used, explaining decisions taken and the certainties involved, this is not possible with a neural network. As indicated above, neural networks process time-dependent spatial patterns and have to be trained rather than programmed.

Just as we have no difficulty recognizing a face, but a great deal of difficulty trying to explain how or why we recognized the face, a trained neural network can recognize the solution to a problem but cannot really explain why. A recent paper by Pau and Götzsche [21] addresses this question. Future AI systems may come to rely on a combination of the experienced neural network to recognize the solution to a problem and an expert system to rationalize the solution given. This combination is termed a cognitive expert system and provides an interesting model of human intelligence.

### APPLICATIONS

### Introduction

In the clinical laboratory, improvements in instrumental systems as a result of the intensive use of microprocessors and better sensors have increased dramatically the rate at which measurement data (both quantitative and qualitative) can be obtained. This has been termed 'data affluence'. However, such complete and complex data do not represent information in a really useful form.

Several tools, including expert systems, are available for the interpretation of data or basic information into useful information, using either conventional data processing or neural networking systems that are adequately trained.

A major distinction can be made between, on the one hand, *stand-alone systems* (examples in table 7) that interact with the user, the main purpose of which is to replace human experts, and *embedded systems* that are integrated into large electrical or mechanical systems and function autonomously.

The classical illustration of this is two different chess-playing systems. The first is a computer program with the potential to beat experts: players communicate with the

*Table 7. Example of stand-alone expert systems used in medicine.*

| Expert system | Domain | Reference |
|---|---|---|
| ABEL | Acid-base electrolytes | Paril *et al.* [34] |
| GARVAN-ES1 | Thyroid | Compton [13] |
| | Colon cancer | Weber *et al.* [67, 68] |
| PONJIP | Blood gas | Leng *et al.* [69] |
| CASNET | Glaucoma | Weiss *et al.* [30] |
| MYCIN | Infectious diseases | Shortliffe [8] |
| INTERNIST-1 | Internal medicine | Miller *et al.* [35] |
| AI/COAG | Haemostasis/coagulation | Lindberg *et al.* [32] |
| MYCIN | Bacterial infection | Buchanon and Shortliffe [29] |
| | Erythrocyte enzymes | Wiener *et al.* [70] |
| cadi-yac | Antibiotic sensitivity | Peyret *et al.* [23] |
| DIAG | Dermatology | Landau *et al.* [56] |
| SESAM-DIABETE | Diabetes | Levy *et al.* [22] |
| MEDICIS | Diagnosis generally | Du Bois *et al.* [11] |
| ESPRE | Transfusion | Sielaff *et al.* [12] |
| OVERSEER | Drug treatment | Bronzino *et al.* [24] |
| Braindex | Brain death | Pfurtscheller *et al.* [71] |
| Audex HM | Audiology instruments | Bonadonna [72] |
| Foetos | Foetal assessment | Alonso-Betanzos *et al.* [73] |

system via a keyboard terminal and text display. The second plays poor chess, but it inspects the board via a TV camera, makes its own moves for itself with a computer driven hand, explains its moves in passable English, improves its play with practice and can accept strategic hints and advice from a tutor. Both of these systems represent fairly advanced projects and both would probably be regarded as intelligent by most people. The stand-alone system is intelligent in a theoretical sense, while the second instrument is an example of what can be called an integrated system with embedded AI. Developing a stand-alone computer expert system that is capable of beating international grand masters would be a major task for the knowledge engineer. However, chess programs operating at a popular level are now fairly widespread. The chess-playing machine would be more challenging in many areas such as image processing, mechanical manipulation, and natural language interfacing.

### Stand-alone systems

Stand-alone expert systems have interactive user interfaces and are designed to be used like experts. Three types of stand-alone systems are discussed below. Expert system development tools have been developed to allow users to build their own knowledge-based systems. Medical diagnosis expert systems are developed using input from domain experts and are designed primarily for interactive use by medical staff and students.

### *Development tools*

An expert system can be built from scratch using a structured language such as Pascal or C, or a fifth generation AI language such as LISP (for example Levy *et al.* [22]) or Prolog (Peyret *et al.* [23], Bronzino *et al.* [24]). In fact, expert systems are eminently suitable for the development of complex decision sequences because the knowledge is separated from the program. Therefore, it is relatively simple to reuse the system shell for a different knowledge base. Despite the development of AI languages that are more efficient in declaring goals and acquiring knowledge, most expert systems are still written in conventional languages (for example C and Fortran).

Many expert system development tools are therefore derived directly from existing expert systems simply by removing the knowledge base. The classic example is the EMYCIN tool developed at Stanford University (Van Melle *et al.* [25]) from the MYCIN expert system. The newer tools for development applications are better than EMYCIN. Using such development tools, rules and decisions can be changed with a minimum of inconvenience and the system iterated until it performs at least as well as the human expert would.

Some examples of these development tools are given in table 8. In some tools, the knowledge base is entered in the form of production rules. In others, the knowledge is

*Table 8. Expert system (language) development tools.*

| Tool | References |
|---|---|
| EMYCIN (Lisp) Stanford | |
| EXPERT (Fortran) Rutgers | Weiss and Kulikowski [74] |
| KEE (Lisp) Intellicorp | Gill *et al.* [75] |
| VP-Expert | O'Connor [9] |
| PC-Plus (Lisp) Texas Instruments | Pfurtscheller *et al.* [78] |
| GoldWorks (Lisp) Bolesian Systems | |
| ART (Lisp) | |
| EXSYS | Marchevsky [76] |
| APEX Fault diagnosis shell | Merry [77] |
| NEXPERT (C) Neuron Data | |
| KAPPA Intellicorp | |
| DELFI (TurboPascal) Delft University | de Swaan *et al.* [78] |
| Pro.M.D. (Prolog) | Pohl and Trendelenburg [79] |

entered either as a matrix of attributes and outcomes from which the system derives rules, as the rules themselves, or as networks of interactions. Purely rule-based systems have the limitation that the structural and strategic concepts must be incorporated in the rules on entry.

The evaluation of tools for developing expert systems is discussed by Rothenberg *et al.* [26]. Predictably, the choice of tool depends on the domain, the problems to be solved and the expertise available. The target environment may impose restraints, for example, the need to integrate the shell into existing hardware and with existing software. Clearly the user interface must be tailored to the user.

Such development tools allow the domain expert, together with the knowledge engineer, to create and debug a knowledge base and integrate it into an expert-system framework that provides the necessary interference engine and user interface.

Software tools for neural net development are also readily available. Collins [19] lists 15 such tools, some of which (for example BrainMaker from California Scientific Software) can be implemented on a standard personal computer.

### *Medical diagnosis*

There is a clear need to be able to deal optimally with the affluence of data and explosion of knowledge still occurring in the medical field. Computer-supported medical decision making (CMD) systems may be the solution and there is evidence that clinical performance may be improved by the use of CMD (see Adams *et al.* [27]).

Perry [28] reviewed knowledge bases in medicine, looking at three basic models. MYCIN and its domain-independent version EMYCIN are rule-based systems and are the basis of expert systems, such as PUFF for lung disease (Buchanon and Shortliffe [29]) and ONCOCIN for cancer therapy (used by Stanford University). Causal models such as CASNET (Weiss *et al.* [30]), that was generalized and extended to form the shell system EXPERT, support expert systems like AI/RHEUM (Porter *et al.* [31]) and AI/COAG (Lindberg *et al.* [32]). Present Illness Program, PIP (Pauker *et al.* [33]), Acid-Base Electrolytes, ABEL (Paril *et al.* [34]), and INTERNIST-1 (Miller *et al.* [35]) are hypothesis-based systems (Bouckaert [36]).

Many medical problems, especially those related only to laboratory data, can be solved using conventional algorithms as they contain data of known imprecision. It has been suggested that only solutions requiring between 10 min and 3 h of clinician time are suitable for expert systems (Frenzel [37]). The upper limit may be the boundary for knowledge representation.

In a review by Winkel [38], 13 expert systems that emphasize the use of laboratory data were listed. Winkel points out that for practical reasons (for example entering data), it may not be meaningful to introduce an expert system into the laboratory or elsewhere unless it is integrated into the hospital or laboratory information system, and this may be a difficult task. The main purpose of the OpenLabs project (with 28 participants from 12 countries) under the European Community's AIM Research and Development Programme is to integrate knowledge-based systems and telematics with laboratory information systems and equipment.

Trendelenburg [39] makes a clear distinction between *internal medicine*, with its large knowledge domains, where there is little computer-supported data flow and not much familiarity with medical computer applications; and *laboratory medicine*, where the domains are clear cut, staff are familiar with medical computer applications and the data flow is computerized. More than 50 European clinical laboratories are applying or developing knowledge-based systems for generating reports on special findings in an instructive and interpretative way. Trendelenburg emphasizes that discussions about the computer-based nature of the reports produced are best avoided, and the responsibility for using the report must be with the user.

Comparisons have been made of the performance of standard computing programs, expert systems and neural networks in medical diagnostic situations. For example, Wied *et al.* [40] discuss the ability of discriminate function analysis and neural networks to classify DNA ploidy spectra. Dawson *et al.* [41] discuss the use of nuclear grade as a prognostic indicator for breast carcinoma. Advances in image analysis can reduce the effect of inter-observer variability. Both Bayesian analysis and neural networking agreed with the human observer for low-grade lesions, whereas nuclear heterogeneity in high-grade tumours resulted in poor agreement (only 20% successful classification). Wolberg and Mangasarian [42] discuss a more successful comparison using 57 benign and 13 malignant samples, in which a multi-surface pattern recognition system scored one false negative, a neural network scored two false positives, and a decision-tree approach scored three false positives. Other examples of neural networks used in medical diagnosis are given in table 9.

A successful neural network will show a balance between the error that it exhibits with its training set and its power to generalize based on the training set. Reduction of training error should thus be treated with caution as overtraining can seriously impair generalization power (Astion *et al.* [43]). It is known that the number and size of hidden neuron layers influences the representation capabilities and the generalization power of the neural network, yet these parameters are often chosen quite arbitrarily.

Expert systems are used widely in clinical laboratories for the validation of biochemical data. For example, Valdiguié *et al.* [44] have used VALAB, an expert system, incorporating 4500 production rules and the forward chaining inference engine KHEOPS, to validate more than 50 000

*Table 9. Examples of neural networks used in medical diagnosis.*

| Domain | Reference |
|---|---|
| Myocardial infarction | Furlong *et al.* [80] |
| ECG | Dassen *et al.* [81] |
| Neonatal chest radiographs | Gross *et al.* [82] |
| Dementia | Mulsant [83] |
| IR spectroscopy | Wythoff *et al.* [84] |

laboratory reports. The system runs as a silent partner in the laboratory organization and is able to dramatically reduce the work involved in the routine validation of data. Neural networks are also being applied to clinical laboratory data. Reibnegger *et al.* [45] show that neural networks are capable of extracting hidden features, but emphasize that validation is even more important when neural networks are used for medical diagnosis based on laboratory information.

### Embedded systems

Embedded knowledge-based systems (i.e. AI systems with no human interaction) are used in laboratory instrumentation at a variety of levels (see table 10). Unfortunately they are not as commonly published as stand-alone systems for medical diagnosis.

The following is a brief description of six different applications of embedded systems that illustrate increasing degrees of compatibility with neural networks; these are: failure detection, user interfacing, image processing, natural language processing, robotics and machine learning.

### *Applications to failure detection, testing and maintenance*

Failure detection, testing and maintenance are knowledge-intensive tasks that rely on experience [46]). Apart from using test procedures, skilled maintenance staff use heuristics and an understanding of how the system works to solve a problem. The high level of performance of maintenance experts suggests that they use powerful hierarchical structures in problem solving. The goal of expert systems in this area is to exploit this procedural knowledge in conjunction with failure detection to generate self-testing procedures and to ease the operator workload in the man–machine interface.

The requirements for an ideal troubleshooting system include minimizing the probability of false alarms and of not detecting failures, minimizing the time taken, effective integration of knowledge, ease of maintenance and expandability, broad application, the ability to handle uncertainties and a large explanation capability.

*Table 10. Typical applications of embedded expert systems.*

* Instrument self testing and diagnosis, including calibration of sensors and displays, trouble shooting of instrument.
* Handling signal acquisition. Transduction of signals from sensors and fast storage of measurement data.
* Treatment of measurement data from signal acquisition and extraction of features for further interpretation. Conversion of data to primary information.
* Image processing in microscopy, for the sorting of biological structures by size, absorption, colour.
* Timing and sequencing of mechanical functions (especially for multichannel analysers). For example keeping track of position sensors, clock timing, parallel movements, and prioritization by queuing theory.
* Storage and retrieval of information for statistics, record comparisons, calibration, standardization.
* Interpretation and presentation of results. Layout of man-machine interface screens, generation and presentation of reports and explanations in customized formats.

A major task is to devise schemes of knowledge representation to allow failure events to be analysed by the merging of very diverse information sources, for example analogue/digital signals, logical variables and test outcomes, texts from verbal reports and inspection images. Embedding the expert systems in a distributed reasoning structure shortens execution times and allows for easy updating.

Groth and Modèn [47] describe a system providing real-time quality control for multitest analysers using a relational data-base management system. This includes a knowledge-based troubleshooter and advisor and was designed for the PRISMA mutichannel analysis system. Another example is Woodbury [48] who developed an expert system for troubleshooting an Hitachi analyser.

### *Image understanding vision system*

Bartels *et al.* [6] describe an image understanding machine vision system for histology which is based on three expert systems. A scene segmentation expert system extracts information from the image. This is an important function and it has been shown [49] that machine vision systems are able to detect subtle differences in morphologic architecture that the human observer is generally unable to discriminate beyond a certain lower limit. The knowledge representation is in the form of a semantic net with associated frames and the knowledge is written in declarative statements. In the case of gland imaging for example, the system might look for a lumen (that is defined in terms of an ellipse ratio of more than 1·50 and an area less than 650 pixels) with glandular nuclei grouped within a certain distance around the periphery (that are defined under object groups) using a statement such as:

Gland IS A SINGLE Lumen WITH
a SET OF Object Groups

The different features found by this system are listed and called by the second interpretative expert system that relates diagnostic conceptual knowledge to them. The 'degree of cibriformity' in prostate glands is, for example, measured by tracing the circumferences of the lumina and relating this to total area to obtain form factor ratio. The structure of the interpretative expert system is very similar to the scene segmentation system.

The third diagnostic expert system is in the form of a spreadsheet, with diagnostic categories, clues, clue values and certainty values. This expert system can run autonomously as an embedded system or can be run interactively as an expert system.

### *User interfaces*

The success of knowledge-based instrumentation systems depends to a great extent on the man-machine interface, both during development when the domain expert and knowledge engineer provide the system with a representation of real knowledge and human reasoning, and later when the user tries to implement the system.

*Table 11. Generic functions for man–machine interfaces (based on Pau [50]).*

* Data handling and presentation (acquisition, coding, compression, presentation, recording)
* Extracting features from measurements for interpretation
* Control of instrument functions and utilities (trouble-shooting and repair are excluded from the man–machine interface)
* Calibration of sensors and displays
* Development of procedures and experiments (for example sequencing experimental steps)
* Generation of reports and explanations
* Retrieval of user information/documentation (for example about the experimental set-up).

Embedded expert systems may not require a user interface. Many embedded systems are controlled by a computer system, or are designed to communicate with other expert systems for example through what is called a 'blackboard' mechanism.

Pau [50] has described generic functions for man-machine interfaces (see table 11). These functions can be backed up by knowledge-based techniques to render them intelligent. Apart from the knowledge bases themselves, such techniques would incorporate search and inference to suggest feasible actions to the user, and rule-based spreadsheets or other presentation systems for display, control and explanation.

The potential of user interfaces goes far beyond simple screen interaction. Tactile and vocal interaction can be added to allow freedom of movement, with a possibility of communication and delegation. This type of system (known as virtual reality) is being developed for commercial purposes, for example in the entertainment industry. It is possible to imagine how such systems could be used for example for training surgeons and there is a challenge now for architects and designers to invent new forms of knowledge representation based on these new technologies that have become available.

*Natural language processing*

Landau *et al.* [51] argue that computer-aided medical diagnosis will not attain widespread acceptance among physicians until it can include automatic speech recognition and proposed a prototype natural language interface. Speech recognition is not just a matter of words in a series. Not surprisingly, intelligent use of language depends on an understanding of the subject. Context, guessed meanings and memories of speech fragments have a lot to do with being able to hear what is being said. This is a pattern-recognition problem and typical of the human brain.

Kohonen [52] developed a phonetic typewriter using a competitive filter neural network. This system achieves 92 to 97% accuracy, with continuous speech and unlimited vocabulary for Finnish and Japanese languages. One hundred words of each new speaker are taken as training samples. The network automatically classifies speech sounds and stores related sounds spatially near each other in the network. The fact that the system does not achieve greater accuracy is due to the many strange rules of languages for example co-articulation of syllables. Japanese and Finnish may actually be rather more logical in this respect than other languages, for example English.

In what might be an imitation of nature, Kurogi [53] used the anatomical and physiological findings on the afferent pathway from the ear to the cortex to develop a neural network that recognizes spoken words, allowing for changes in pitch, timing etc.

*Robotics*

The mechanical functions of a robot are like a human is, but remain relatively unintelligent if the robot does not include sensor or recognition systems that can allow it to know its environment. Robots are particularly useful for control and handling of samples in sterile or hazardous environments, in situations where a human operator either cannot or should not be present [3].

Robotic arms are usually programmed to perform one specific task at a time. The intelligent robotic arm would be able to move across a variety of pathways carrying a variety of loads. The adaptive robotic arm is a typical application area for expert systems and neural networks. For example, Kawato and coworkers in Osaka have developed an adaptive robot arm controller based on a model of human motor control and neural networks, having three degrees of freedom.

*Machine learning*

As early as 1950, Turing [54] foresaw the potential of systems capable of autonomous learning and admitted that he would regard a machine that could program itself as intelligent.

Bartels *et al.* [55] discuss the issues involved in the design of autonomous-learning expert systems in histopathology. The autonomous learning process must have a built-in control function to keep the process goal-oriented (and by implication the process must have a goal and criteria defined by which progress towards the goal can be assessed).

A decisive element in expert systems is the structuring of knowledge and the interrelationships between knowledge components. In histopathology, facts are obtained from the human observer or from the image processing and image-extraction programs. One type of learning consists of updating a knowledge base with new blocks of data that can fine tune or stabilize decision rules. However, real autonomy is first reached in systems that can augment their own rule sets or, like neural networks, are self-organizing.

### CONCLUSIONS

### Introduction

This review has looked at the development of programmed software, the flexibility this has allowed in system operation and control, and how this has led to intelligent clinical laboratory systems.

The knowledge base of expert systems contains the collective expertise of domain experts (in the clinical laboratory and related medical domains) and the knowledge engineers who represent this expertise. An unresolved question concerns the ownership and exploitation rights of such knowledge bases.

Since this expertise will be used to guide medical decision making, it will be subject to regulation by the appropriate authorities. Just like any other medical device, expert systems need to be evaluated and documented. Responsibility for the consequences of any inappropriate use of knowledge bases and AI rests with the professional user of such systems.

### *Regulatory affairs*

Kahan [56] has reviewed US Food and Drug Administration (FDA) regulation of medical device software. In the US, the FDA regulates medical device software via a draft GMP Guidance for software [57] and a draft reviewer guidance [58]. This is supported by a technical report [59] used by FDA investigators.

The 1987 Draft Policy on Software [60] is applied to computer products that are intended to affect the diagnosis and treatment of patients. It was intended to be primarily applicable to stand-alone medical devices, rather than to software that is a component of a medical device (embedded). It does apply to some stand-alone AI products now entering the US market. For example, in 1988 the FDA decided to include stand-alone software for blood banks under regulation (these were previously exempt). The 1987 Draft Policy will probably not be finalized and stand-alone software will probably be treated as exempt from premarket approval except when a risk is perceived.

Embedded systems are a more difficult question. In November 1990 a Canadian company issued a safety bulletin because their embedded software to calculate patient dose in radiation therapy was flawed. This sharpened FDA interest. The draft GMP guidance for software is specific about the analysis of software and for example, stipulates that software functions used in a device must perform as intended and testing should verify this. Such software must be tested separately by simulation testing in the environment in which the software will be used.

The Health Industry Manufacturers' Association (HIMA) has expressed concern about the FDA Guidance—for example arguing that source codes should not be copied during inspection by FDA and confidential algorithm specifications should not be revealed.

In Europe, a recent EC Medical Device Directive to Trade Associations includes software in the definition of medical devices for the first time. Previously the definition only included software that was incorporated into a device, but now the definition includes software itself (which presumably means stand-alone systems). This has caused some confusion among European manufacturers.

In May 1991 the EC issued a Directive [61] on the legal protection of computer programs, requiring member states to protect such programs by copyright under the Berne Convention for the Protection of Literary and Artistic Works.

### Evaluation of expert systems

Miller [62] distinguishes three levels of evaluation of expert systems; evaluation of research contribution of a development prototype, validation of knowledge and performance and evaluation of the clinical efficacy of an operational system.

Evaluation of developmental prototypes is an iterative process and the requirements of a particular system will change as the system matures towards the final operational system. One of the most interesting questions is how to evaluate and limit a neural network that can continue to learn.

Knowledge should be assessed in terms of accuracy, completeness and the consistency and performance of the system matched with that of experts. A critical aspect is the capacity of the system to know its own limitations and, for example, to fail to make a diagnosis when presented with an unfamiliar case. At best expert systems used to perform just as well as the experts themselves (Yu *et al.* [63], Quaglini and Stefanelli [64]). This situation is changing as validation, verification and maintenance tools are introduced (Pau [14]).

Evaluation of an AI system in operation depends on the domain and the clinical role it is expected to play. There are a number of issues of interest that are very difficult to assess, for example the impact of the system on the quality of health care and the user's subjective reaction to the system. Mainly because of this there are few evaluations of operational systems.

### The need for transparency

We would like greater transparency in the application of artificial intelligence in the clinical laboratory. It is particularly difficult in the clinical laboratory environment to evaluate embedded expert system as they generally appear as black boxes to the user. Wulkan [7] asks the question who is to be held responsible if the system contributes with faulty information that could result in a decision that ultimately proves fatal to the patient? Hoffmann [65] suggests that responsibility should lie with those who apply such systems to provide information for the clinic.

There is a danger that expert systems may lend authority and even prejudices to shallow decisions by not disclosing the exact nature of the inference mechanisms, not identifying the human experts who have provided the expertise or the knowledge engineers who have interpreted this expertise for the expert system, or not disclosing the learning set used to train a neural network. Neural networks in particular are less amenable to logical explanation, although they can be evaluated in terms of the training data and the network performance on test data (Hart and Wyatt [66]). There are positive signs that this situation is beginning to change, with the introduction of explanation facilities that are also implemented on neural networks (Pau [14]).

It would seem ironic if the mechanisms of artificial intelligence were applied ineffectively, indeed unintelligently, to clinical laboratory information. Concerns have been expressed that applications of artificial intelligence could lead to a false uniformity and discourage dissenting opinion by crystallizing the majority opinion and current state of the art in a more permanent form. Again the situation seems to be improving and it now seems more likely that minority opinions can be included in the accumulation of incremental knowledge as the knowledge base is built up.

We believe that it would be useful to look at the knowledge base in an AI system as a form of 'collective intellectual property' and to elaborate a set of recommendations for its exploitation.

### Items for future debate

This review could be supplemented with a survey, in the form of a questionnaire, concerning applications of embedded systems in laboratory instrumentation.

We recommend that a Working Group should be set up to draft guidelines for applications of artificial intelligence in the clinical laboratory. For expert systems, recommendations should be considered for the identification of the domain experts who have contributed to the expert system, the representation used (and its authorship) and the inference strategy used. For neural networks, we believe that the Working Group should consider recommendations for the training data and network performance on test data.

In addition, we would like to see the Working Group debate the question of intellectual property rights of authors of expert systems and additional knowledge that may be added to an existing knowledge base. Finally, the Working Group might debate the question of copy protection of commercial systems in relation to publication and the need for greater transparency.

### References

1. Büttner, J. and Habrich, C., *XIII International Congress of Clinical Chemistry.* (GIT Verlag, Darmstadt, 1987).
2. Skeggs, L. T., *Analytical Chemistry*, **38** (1966), 31A.
3. Ozawa, L., Schnipelsky, P., Pardue, H. L., Place, J. F. and Truchaud, A., *Annales de Biochimie Clinique*, **49** (1991), 528.
4. Pardue, H. L., Ozawa, K., Place, J. F., Schnipelsky, P. and Truchaud, A., Systematic top-down approach to clinical chemistry *Annales de Biologie Clinique* **52** (1994) 311–320.
5. Caudill, M. and Butler, C., *Naturally Intelligent Systems* (MIT Press, Boston, 1990).
6. Bartels, P. H. and Weber, J. E., *Analytical and Quantitative Cytology and Histology*, **11** (1989).
7. Wulkan, R. W., Thesis: Expert systems and multivariate analysis in clinical chemistry (Erasmus University, Rotterdam 1992).
8. Shortliffe, E. H., *Computer-Based Medical Consultations: MYCIN* (Elsevier, New York, 1976).
9. O'Connor, M. L. and McKinney, T., *Archives of Pathology and Laboratory Medicine*, **113** (1989), 985.
10. Bartels, P. H., Bibbi, M., Graham, A., Paplanus, S., Shoemaker, R. L. and Thompson, D., *Analytical Cellular Pathology*, **1** (1989), 195.
11. Du Bois, P., Hermanus, J. P. and Cantraine, F., *Medical Informatics*, **14** (1989), 1.
12. Sielaff, B. H., Connelly, D. P. and Scott, E. P., *IEEE Transactions on Biomedical Engineering*, **36** (1989), 541.
13. Compton, P., *Clinical Chemistry*, **36** (1990), 924.
14. Pau, L. F., *Guidelines for Prototyping, Validation and Maintenance of Knowledge Based Systems Software* (Electromagnetics Institute, Technical University of Denmark, 1990).
15. Tesler, L. G., *Scientific American*, **265** (1991), 54.
16. Aleksander, I., In M. Reeve and S. E. Zenith (Eds.), *Parallel Processing and Artifical Intelligence* (John Wiley & Sons, Chichester 1989).
17. Fornili, S. L., *Research and Development* (Cahners Publishing, Hoofddorp, 1989).
18. McCulloch, W. S. and Pitts, W., *Bulletin of Mathematical Biophysics*, **5** (1943), 115.
19. Collins, M., *Bio/technology*, **11** (1993), 163.
20. Psaltis, D., Brady, D., Gu, X. G. and Lin, S., *Nature*, **343** (1990), 325.
21. Pau, L. F. and Götzsche, T., *Journal of Intelligent and Robotic Systems* (April 1992).
22. Levy, M., Ferrand, P. and Chirat, V., *Computers in Biomedical Research*, **22** (1989), 442.
23. Peyret, M., Flandrois, J. P., Carret, G. and Pichat, C., *Pathologic Biologie*, **37** (1989), 624.
24. Bronzino, J. D., Morelli, R. A. and Goethe, J. W., *IEEE Transactions on Biomedical Engineering*, **36** (1989), 533.
25. Van Melle, W., Shortliffe, E. H. and Buchanan, B. G., *Machine Intelligence*, Infotech State of the Art Report, Series 9 (1989), No. 3.
26. Rothenberg, J., Paul, J., Kameny, I., Kipps, J. R. and Swensson, M. *Evaluating Expert System Tools. A Framework and Methodology* (The RAND Corporation, Santa Monica, 1989).
27. Adams, I. D., Chan, M. and Clifford, P. C., *British Medical Journal*, **293** (1986), 800.
28. Perry, C. A., *Bulletin of the Medical Libraries Association*, **78** (1990), 271.
29. Buchanon, B. G. and Shortliffe, E. H., (Eds.) *Rule Based Expert Systems: The MYCIN Experiments of the Stanford Heuristic Programming Project* (Addison-Wesley, Stanford, 1984).
30. Weiss, S. M., Kulikowski, C. A., Amarel, S. and Safir, A., *Artificial Intelligence*, **11** (1978), 145.
31. Porter, J. F., Kingsland, L. C., Lindberg, D. A. B. and Shah, I., *Arthritis and Rheumatism*, **31** (1988), 219.
32. Lindberg, D. A. B., Gaston, L. W., Kingsland, L. C. and Vanker, A. D., In *Proceedings of the Fifth Annual Conference on Computer Applications in Medical Care* (November 1981).
33. Pauker, S. G., Garry, G. A., Kassirer, J. P. and Schwarz, W. B., *American Journal of Medicine*, **60** (1976), 981.
34. Paril, R. S., Szolovits, P. and Schwartz, W. B., In Szolovits, P. (Ed.) *Artifical Intelligence in Medicine* (Westview Press, Boulder, 1982).
35. Miller, R. A., Pople, H. E. and Myers, J. D., *New England Journal of Medicine*, **307** (1982), 468.
36. Bouckaert, A., *International Journal of Biomedical Computing*, **20** (1982), 123.
37. Frezel, L. E., *Understanding Expert Systems* (Howard W. Sams & Co., Indianapolis, 1987).
38. Winkel, P., *Clinical Chemistry*, **35** (1989), 1595.
39. Trendelenburg, C. and Pohl, B., *Anales de Biologie Clinique*, **51** (1993), 226.
40. Wied, G. L., Bartels, P. H., Bibbo, M. and Dytch, H. E., *Human Pathology*, **20** (1989), 549.
41. Dawson, A. E., Austin, R. E. and Weinberg, D. S., *American Journal of Clinical Pathology*, **95** (1991), 29.
42. Wolberg, W. H. and Mangasarian, O. L., *Analytical and Quantitative Cytology and Histology*, **12** (1990), 314.
43. Astion, M. L., Wener, M. H., Thomas, R. G., Hunder, G. G. and Bloch, D. A., *Clinical Chemistry*, **39** (1993), 1998.

44. Valdiguié, P. M., Rogari, E. and Philippe, H., *Clinical Chemistry*, **38** (1992), 83.
45. Reibnegger, G., Weiss, G., Werner-Felmayer, G., Judmaier, G. and Wachter, H., *Proceedings of the National Academcy of Sciences*, **88** (1991), 11426.
46. Pau, L. F., *Expert Systems*, **3** (1986), 100.
47. Groth, T. and Modèn, H., *Computer Methods and Programs in Biomedicine*, **34** (1991), 175.
48. Woodbury, W. F., *Laboratory Medicine*, **20** (1989), 176.
49. Paplanus, S. H., Graham, A. R., Layton, J. M. and Bartels, P. H., *Analytical and Quantitative Cytology and Histology*, **7** (1985), 32.
50. Pau, L. F., *Jinko Chino Gakkaishi*, **5** (1990), 106.
51. Landau, J. A., Norwich, K. H. and Evans, S. J., *International Journal of Bromedical Computing*, **24** (1989), 117.
52. Kohonen, T., In I. Aleksander (Ed.), *Neural Computing Architectures* (MIT Press, Harvard, 1989).
53. Kurogi, S., *Biol. Cybern.*, **64** (1991), 243.
54. Turing, A. M., *Mind*, **59** (1950), 433.
55. Bartels, P. H., Weber, J. E. and Duckstein, L., *Analytical and Quantitative Cytology and Histology*, **10** (1988), 299.
56. Kahan, J. S., *Clinica*, **463** (1990), 14.
57. *Medical Device GMP Guidance for Investigators: Application of Medical Device GMPs to Computerized Devices and Manufacturing Processes* (US FDA Draft, November 1990).
58. *Reviewer Guidance for Computer Controlled Medical Devices undergoing 510(k) Review* (US FDA, Second Draft, February 1991).
59. *Software Development Activities—Reference Materials and Training Aids for Investigators* (Office of Regulatory Affairs, US FDA; July 1987).
60. *Draft Policy for the Regulation of Computer Programs* (US FDA, reissued November, 1989).
61. *European Council Directive 91/250/EEC* (May 1991).
62. Miller, P. L., *Computer Methods and Programs in Biomedicine*, **22** (1986), 5.
63. Yu, V. L., Fagan, L. M. and Wraith, S. M., *Journal of the American Medical Association*, **242** (1979), 1279.
64. Quaglini, S. and Stefanelli, M., *Computers in Biomedical Research*, **21** (1988), 307.
65. Hoffmann Arbeitsgruppe Geräte-evaluation: Interaktion zwischen mechanisierten Analysegeräten und wissensfor-arbeitenden Systemen. *Mitteiling Deutsche Gesellschaft für Klinische Chemie*, **1** (1990), 25.
66. Hart, A. and Wyatt, J., *Medical Informatics*, **15** (1990), 229.
67. Weber, J. E., Bartels, P. H., Griswold, W., Kuhn, W., Paplanus, S. H. and Graham, A. R., *Analytical and Quantitative Cytology and Histology*, **10** (1988), 150.
68. Weber, J. E. and Bartels, P. H., *Analytical and Quantitative Cytology and Histology*, **11** (1989), 249.
69. Leng, Y. W., Pau, L. F., Sigaard Andersen, O. and Gøthgen, I. H. *Proceedings A.A.A.I. Symposium*, AI in Medicine, (Stanford, CA, March 1990).
70. Wiener, F., de Verdier, C.-H. and Groth, T., *Scandinavian Journal of Clinical Laboratory Investigation*, **50** (1990), 247.
71. Pfurtscheller, G., Schwarz, G., Moik, H. and Haase, V., *Biomed. Tech. Biomedicinische Technik*, **34** (1989), 3.
72. Bonadonna, F., *Medical Informatics*, **15** (1990), 105.
73. Alonso-Betanzos, A., Moret-Bonillo, V. and Hernadez-Sande, C., *IEEE Transactions on Biomedical Engineering*, **38** (1991), 199.
74. Weiss, S. M. and Kulikowski, C. A., In *Proceedings Sixth International Conference on Artifical Intelligence* (Information Processing Society of Japan, Tokyo, 1979).
75. Gill, H., Ludwigs, U., Matell, G., Rudowski, R., Shahsavar, R., Strom, C. and Wigertz, O., *Int. J. Clin. Monit. Comput.*, **7** (1990), 1.
76. Marchebsky, A. M., *Analytical and Quantitative Cytology and Histology*, **13** (1991), 89.
77. Merry, M., *GEC Journal of Research* **1** (1983), 39.
78. de Swaan, A. H., Groen, A., Hofland, A. G. and Stoop, J. C., *Delft Progress Report*, **9** (1984), 63.
79. Pohl, B. and Trendelenburg, C., *Methods of Information in Medicine*, **27** (1983), 111.
80. Furlong, J. W., Dupuy, M. E. and Heinsimer, J. A., *American Journal of Clinical Pathology*, **96** (1991), 134.
81. Dassen, W. R., Mulleneers, R., Smeets, J., den Dulk, K. and Cruz, F., *Journal of Electro-cardiology* **23** (1990), 200.
82. Gross, G. W., Boone, J. M., Greco-Hunt, V. and Greenberg, B., *Invest. Radiol.*, **25** (1990), 1017.
83. Mulsant, B. H., *MD Comput.*, **7** (1990), 25.
84. Wythoff, B. J., Levine, S. P. and Tomellini, S. A., *Analytical Chemistry*, **62** (1990), 2702.

